# Insufficient fibrinogen response following free flap surgery is associated with bleeding complications

**DOI:** 10.3205/iprs000101

**Published:** 2016-11-22

**Authors:** Jonas Kolbenschlag, Yannick Diehm, Adrien Daigeler, David Kampa, Sebastian Fischer, Nicolai Kapalschinski, Ole Goertz, Marcus Lehnhardt

**Affiliations:** 1 Department of Plastic Surgery, Reconstructive and Esthetic Surgery, Handsurgery, Martin-Luther-Hospital Berlin, Germany; 2 Department of Plastic Surgery, Burn Center, Sarcoma Center, BG University Hospital, Ruhr University, Bochum, Germany; 3 Department of Hand-, Plastic and Reconstructive Surgery, Burn Trauma Center, BG Trauma Center Ludwigshafen, University of Heidelberg, Ludwigshafen, Germany; 4 Department of Surgery, BG University Hospital Bergmannsheil, Ruhr University, Bochum, Germany

**Keywords:** coagulation, free flap, microsurgery, bleeding, platelet, fibrinogen

## Abstract

**Background: **Microvascular tissue transfer has become a safe and reliable tool in the reconstructive armamentarium, yielding high success rates. However, little is known about the changes in coagulation after free tissue transfer and their potential impact on morbidity.

**Methods: **Fibrinogen concentration and platelet count among other values were available and assessed in 139 undergoing free tissue transfer before, immediately after, and 1–3 as well as 8–11 days after surgery. In patients undergoing urgent revision for either bleeding or microvascular thrombosis, blood samples were drawn directly before re-exploration.

**Results:** In the patients without any surgical revision and in those with thrombosis of the microvascular pedicle, both fibrinogen concentration and platelet count increased significantly during the early and late post-operative window. Patients that developed bleeding necessitating re-exploration showed an inadequate increase in fibrinogen levels, resulting in significantly lower concentrations compared to the other two groups. There were no significant differences in platelet count or PTT between these groups.

**Conclusion:** Free flap surgery induces acute and subacute changes in coagulation, comparable to other major surgeries and severe injuries. This leads to an increase in platelet count and fibrinogen over the post-operative course. Patients that developed bleeding requiring surgical re-exploration showed an insufficient increase in fibrinogen, resulting in significantly lower fibrinogen levels. Therefore, monitoring and correction of fibrinogen levels might aid in preventing or treating bleeding complications following free flap surgery.

## Zusammenfassung

**Hintergrund:** Mikrovaskulärer Gewebetransfer stellt ein sicheres und zuverlässiges Werkzeug im rekonstruktiven Armamentarium dar. Bezüglich der postoperativen Veränderungen der Gerinnung nach freiem Gewebetransfer und dessen potentieller Morbidität gibt es jedoch nahezu keine Daten.

**Methoden:** Plasma-Fibrinogenkonzentration und Thrombozytenzahl, zusammen mit anderen Parametern, wurden bei 139 Patienten, welche einen freien Gewebetransfer erhielten, vor dem Eingriff, unmittelbar danach und am 1.–3. und 8.–11. postoperativen Tag bestimmt. Im Falle einer Revision wurden die Werte unmittelbar vor dem Revisionseingriff abgenommen.

**Ergebnisse:** Bei Patienten ohne chirurgische Revisionen und bei denen mit Revision wegen Thrombosen des Gefäßstiels stiegen Fibrinogenkonzentration und Thrombozytenzahl während beider postoperativer Zeitpunkte signifikant an. Bei Patienten mit Revision wegen Blutung war kein signifikanter Fibrinogenanstieg zu verzeichnen, so dass deren Wert deutlich niedriger war. Bezüglich der Thrombozytenzahl und der PTT ergaben sich keine Unterschiede zwischen den Gruppen.

**Schlussfolgerung:** Mikrovaskulärer Gewebetransfer führt zu akuten und subakuten Veränderungen der Blutgerinnung, vergleichbar mit anderen ausgedehnten Operationen oder Traumata. Diese führen zu einem Anstieg von Fibrinogen und Thrombozyten. Bei Patienten mit revisionspflichtigen Blutungen zeigte sich ein unzureichender Fibrinogenanstieg. Ein Monitoring und gegebenenfalls die Korrektur der Fibrinogenwerte könnte daher potentiell helfen, Blutungskomplikationen nach freien Lappenplastiken vorzubeugen oder diese zu behandeln.

## Introduction

Microvascular tissue transfer has become a safe and reliable tool in the reconstructive armamentarium, yielding high success rates [1]. However, several influencing factors can still deteriorate the outcome. For example, hypercoagulable states seem to be associated with thrombosis of the microvascular pedicle and subsequent flap loss [2], [3], [4], [5], [6]. 

On the other hand, postoperative bleeding can lead to hematoma formation, potentially compromising flap perfusion as well as necessitating the transfusion of blood products, which can further worsen outcomes [7], [8], [9]. Most of the available literature regarding the influence of coagulation on microvascular thrombosis and hematoma formation is based on the identification of preoperative markers [2], [10], [11], [12]. Such an approach is most reasonable, since at that point in time, these factors might still be approachable by therapeutic interventions. However, the coagulation system is dynamically and known to undergo significant changes after trauma or major surgery [13], [14], [15], [16], [17]. There is limited insight into the natural course of these changes over time following major surgery and, to our knowledge, no reports in patients undergoing microvascular reconstruction [18]. Also, the ability to identify patients which are at higher risk to develop postoperative bleeding might lead to reduced transfusion rates, mitigating the associated morbidity and costs [7]. We therefore sought to investigate the short-term changes in coagulation of patients after free flap transfer and their potential influence on microvascular thrombosis and hematoma formation.

## Patients and methods

This study was conducted in accordance to local regulation and the declaration of Helsinki. 197 consecutive patients undergoing free tissue transfer at our institution were retrospectively identified. A review of their medical charts including surgery- and patient profil-related data as well as laboratory results was conducted. Blood was drawn between 12 and 24 hours before surgery for the perioperative values and at the induction of anesthesia for patients undergoing urgent revision (bleeding or thrombosis of the pedicle). For the patients without revision surgery, laboratory analyses drawn in an early (1–3 days) and late (8–11) days postoperative window were evaluated to assess for the natural course of coagulation over time. One-way ANOVA with post-hoc Tukey’s analysis was performed to assess differences between groups. Paired t-tests were used to assess differences at different points in time within one group. A p-value of <0.05 was considered to be statistically significant. Data is presented as mean values ± SD. 

## Results

The average age at the time of operation was 50.4±14 years, 60% patients were male. The reconstructed defects were associated with trauma in 60% of cases and with cancer resection in 25% of cases. They were predominantly located on the lower extremity (60%), trunk (22%) and upper extremity (16.5%).

Out of the 197 patients, 15 underwent urgent reoperation for bleeding (7.5%) and 27 patients for thrombosis of the microvascular pedicle (13.5%) within the first 72 postoperative hours. There were no donor-site hematomas requiring immediate re-exploration and no bleeding incidents more than 72 hours after surgery.

Current laboratory analyses could be obtained for all patients undergoing urgent revisions (bleeding and thrombosis) and for 97 patients without the need for revision surgery, which served as controls. Therefore, complete laboratory analyses at the respective time points were available for 139 out of the 197 screened patients. The distribution of post-operative anti-coagulant drugs (low molecular heparin vs. unfractioned heparin was not significantly different between the groups (p>0.05), neither was the partial thromboplastin time (PTT) at the time of revision (bleeding: 43±6 s vs. thrombosis: 42.9±9 vs. control: 47.6±15, p>0.05).

### Course over time without revision

Mean preoperative platelet count in patients without revisions was 346.6±127 thsd/µl. 

It showed a significant decrease to 280.1±121 thsd/µl during the immediate postoperative phase (p<0.01 vs. preoperative values) and a further decrease to 259.3±104 thsd/µl in the early post-operative period (p=0.3 vs. immediately post-op). In the late post-operative period, platelet counts rose to 448±169 thsd/µl (p<0.001). 

**Figure 1:**
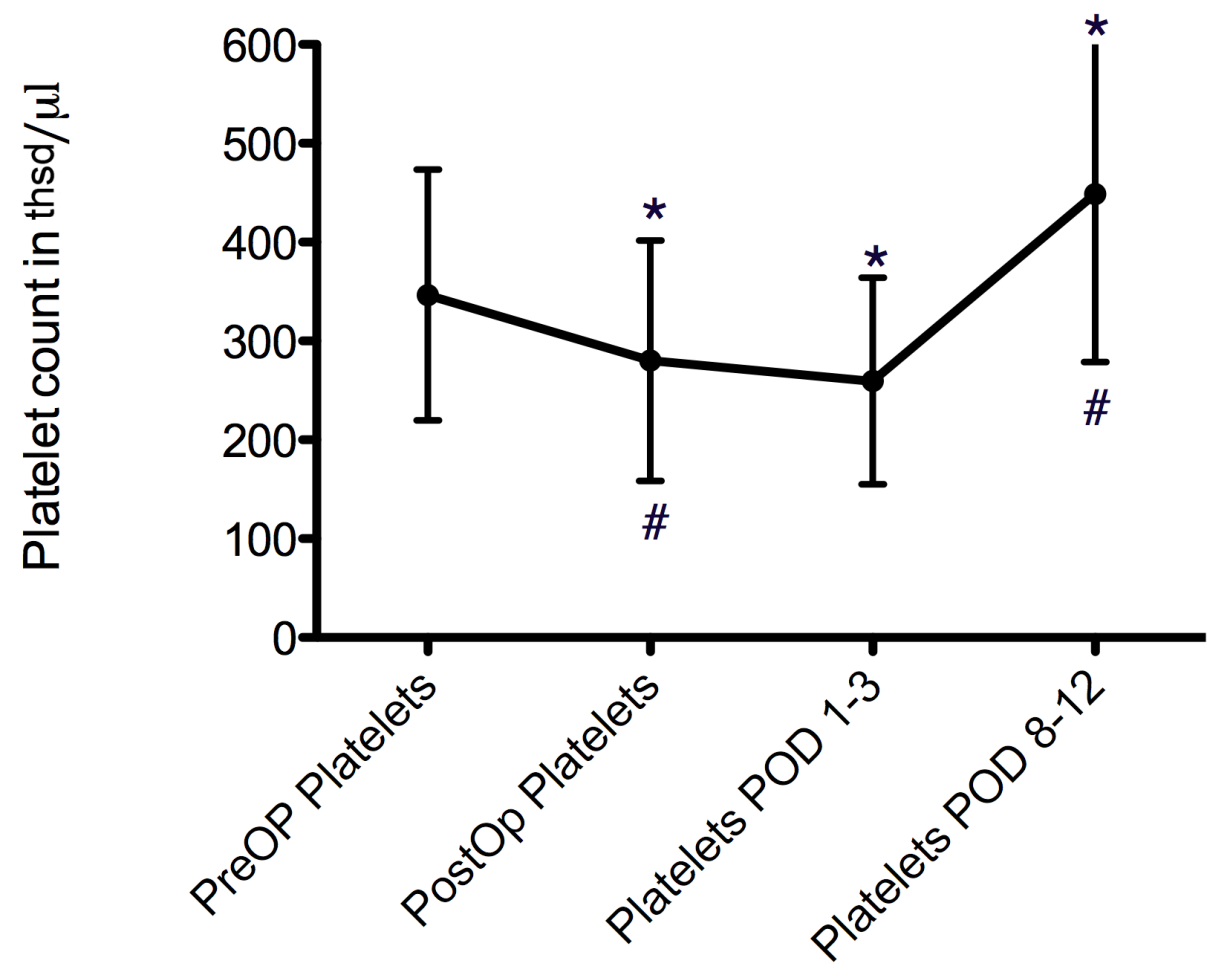
Course of platelet count over time, given as mean values ± SD. * marks significant differences when compared to preoperative values. # marks significant differences compared to the prior point in time.

Also, each point in time was statistically significant different from the preoperative values (p<0.03 for all; Figure 1 (Fig. 1)). 

**Figure 2:**
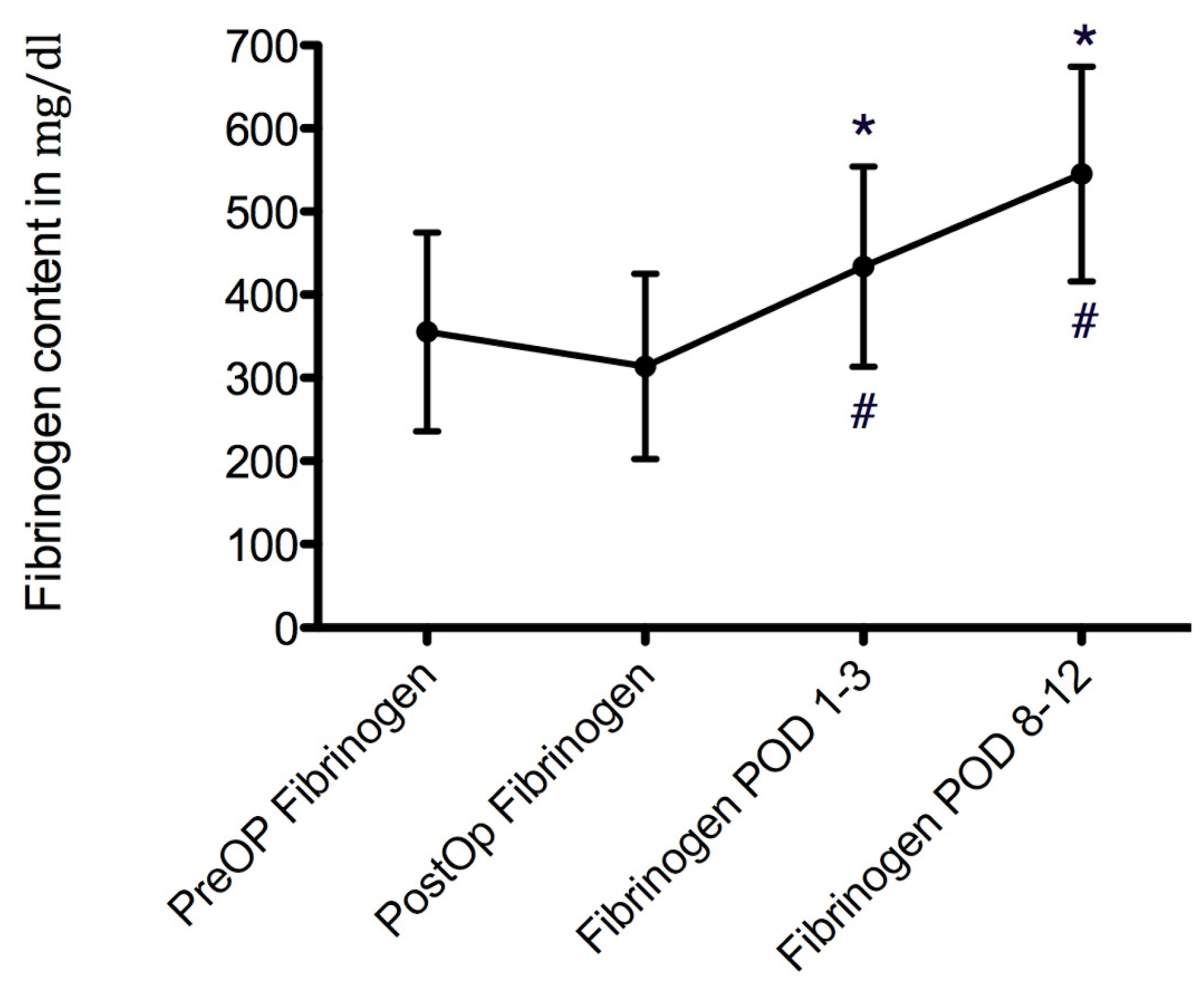
Course of fibrinogen values over time, given as mean values ± SD. * marks significant differences when compared to preoperative values. # marks significant differences compared to the prior point in time.

Mean preoperative fibrinogen values in patients without revisions were 355.4±119 mg/dl. It declined to 313.8±111 mg/dl in the immediate postoperative phase (p=0.2) and then increased to 433±120 mg/dl in the early postoperative phase (p<0.001 vs. immediate postoperative phase). Fibrinogen values further increased over time, up to 545.3±129 mg/dl during the late postoperative period (p=0.002). In addition, the values at the two last points in time were significantly higher than the preoperative values (p<0.001 for both, Figure 2 (Fig. 2)).

### Thrombosis

In the 27 patients that developed thrombosis of the microvascular pedicle within the first 72 hours after surgery, the mean platelet preoperative platelet count was 334.3±100 thsd/µl. This was not significantly different from the preoperative values with patients without revisions and patients that developed postoperative bleeding (p>0.05 for both). It declined to 249.3±59 thsd/µl in the early postoperative phase, resulting in a significant difference compared to preoperative values of patients without revisions (p<0.05). The difference to the preoperative values of patients with thrombosis was not statistically significant (p>0.05).

The mean preoperative fibrinogen content for these patients was 381.7±100 mg/dl, there were no significant differences between the three groups. It increased significantly to 446.3±126 mg/dl in the early post-operative period compared to baseline values (p=0.04).

### Bleeding

Fifteen patients developed a hematoma formation at the recipient site that required urgent reoperation within the first 72 postoperative hours. The mean preoperative platelet count for these patients was 280±109 thsd/µl, unsignificantly lower than in the other two groups. It decreased significantly to 207±70 thsd/µl in the early postoperative period (p=0.03). 

Mean preoperative fibrinogen values were 316.4±32 mg/dl, which slightly increased to 338.42±42 mg/dl in the early postoperative phase (p=0.6). Due to the more pronounced increases in fibrinogen in the groups without any revision and the patients that developed thrombosis, the fibrinogen values in this group were significantly lower in the early postoperative phase (p=0.03).

**Figure 3:**
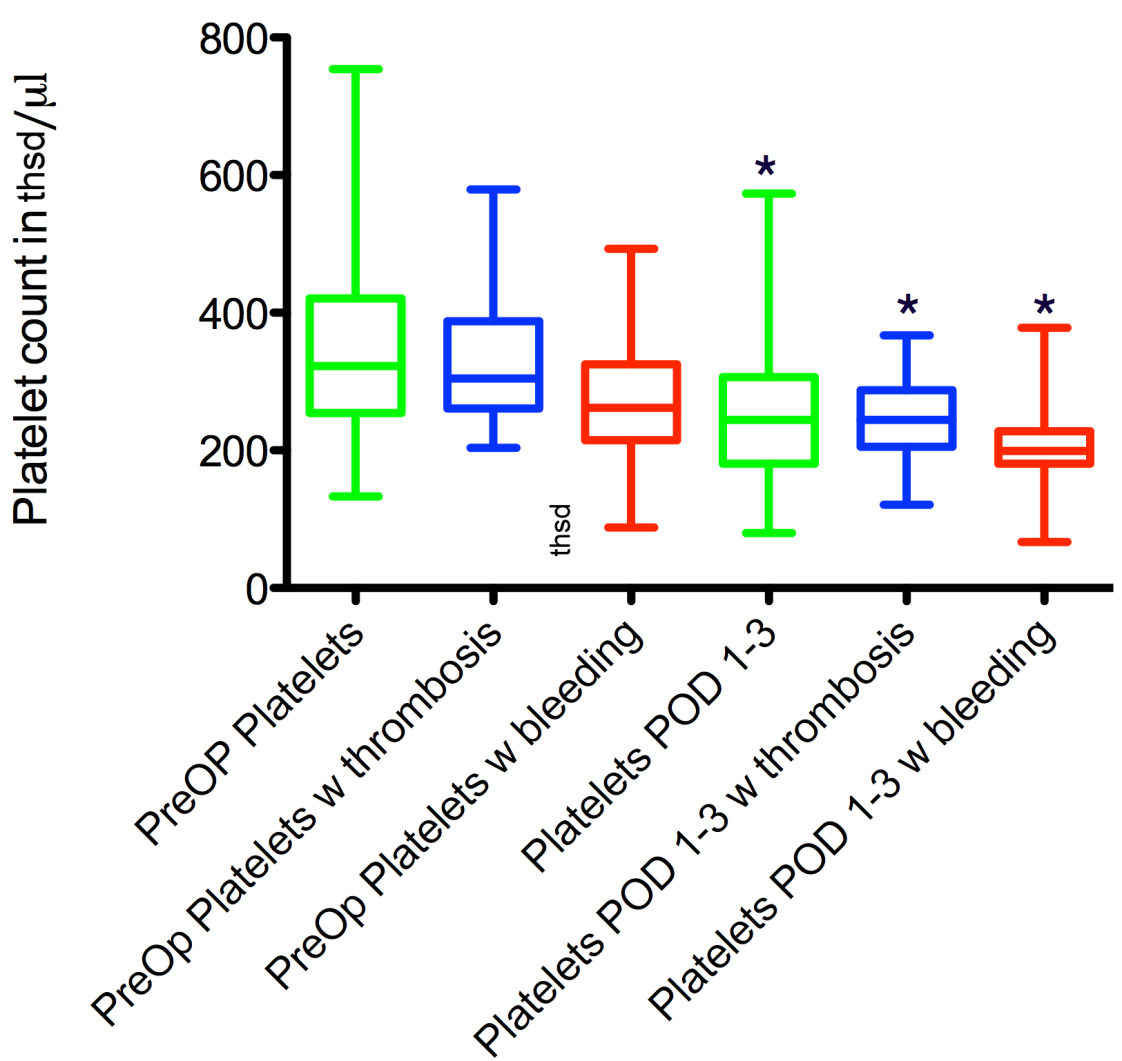
Comparison of pre- and early post-operative platelet counts in patients without revision and with revision for bleeding and thrombosis. * marks a significant difference to the preoperative values. # marks a significant difference compared to the other groups at the same point in time.

**Figure 4:**
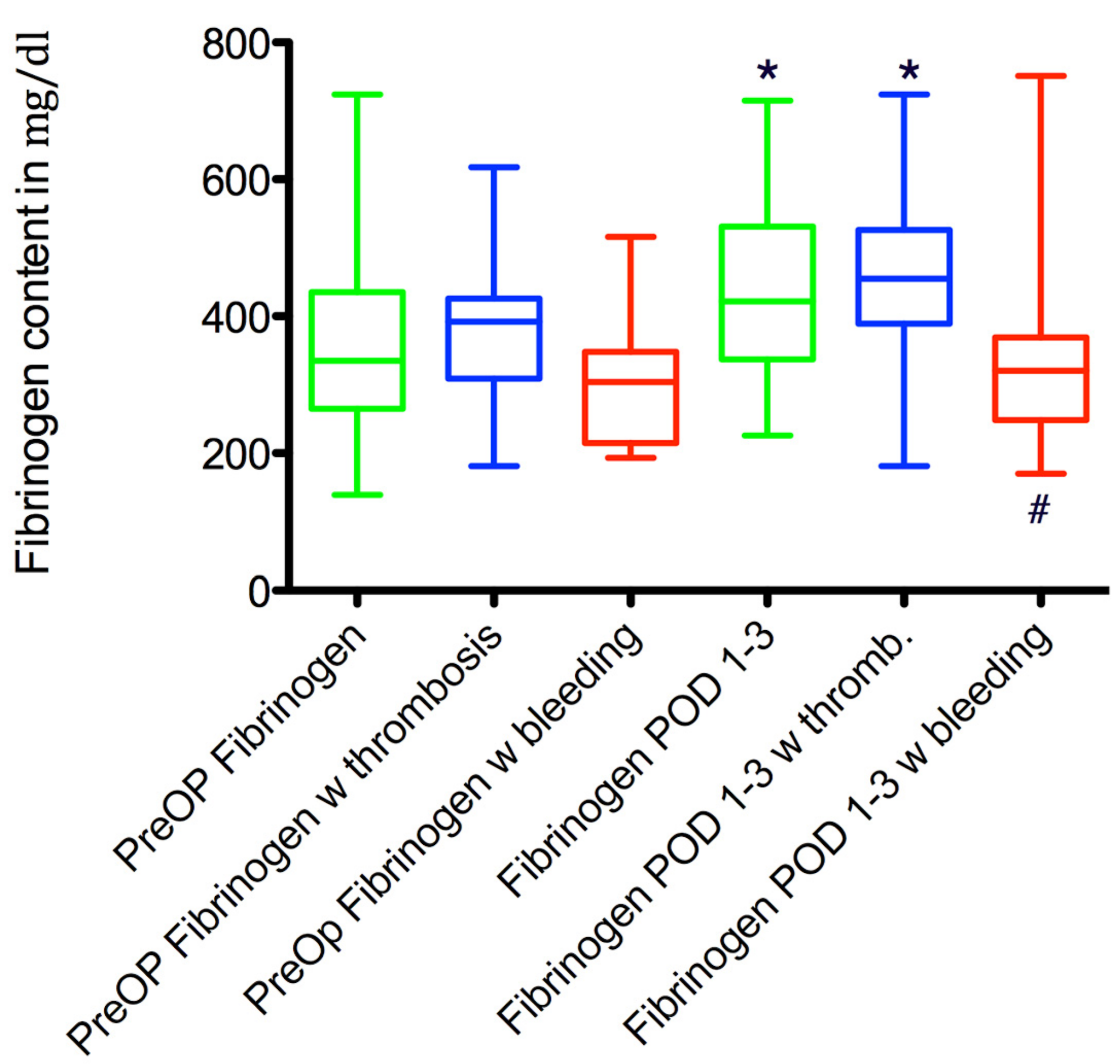
Comparison of pre- and early post-operative fibrinogen levels in patients without revision and with revision for bleeding and thrombosis. * marks a significant difference to the preoperative values. # marks a significant difference compared to the other groups at the same point in time.

See Figure 3 (Fig. 3) and Figure 4 (Fig. 4) for a summary of these findings.

## Discussion

In patients without the need for surgical revision in the early postoperative phase, both platelet count and fibrinogen content increased over the postoperative period. However, while the platelets showed an initial decrease immediately postoperatively and during the early phase, fibrinogen content decreased only insignificantly during the operation. This might partly be explained by intra-operative blood loss and thrombus formation, but also hemodilution due to volume resuscitation. A similar increase in platelet count and fibrinogen can be seen after other major surgeries or trauma, enabling the organism to deal with injuries by sealing off vessels, thus reducing blood loss [16], [19]. Due to such changes, not only the preoperative coagulation status might impact bleeding or thrombosis of the microvascular pedicle [2]. However, the patients that developed such thrombotic events did not significantly differ from the control group in terms of absolute values of fibrinogen and platelets nor their course over time. This might be explained by the fact that most thrombotic events of the microvascular occur due to technical difficulties or direct tissue damage. This is consistent with prior reports, in which a hypercoagulable state was not predictive of pedicle thrombosis in general but of recurrent events leading to thromboembolic flap loss [2]. Therefore, the choice of anti-coagulatory therapy can most likely be chosen according the preoperative risk-profile of the individual patient. 

In patients developing bleeding at the recipient site that required surgical re-exploration however, the fibrinogen values did not increase significantly, resulting in significantly lower fibrinogen concentrations in these patients. Such an inadequate response can be due to various factors. For one, ongoing hemodilution due to volume resuscitation might lead to dimished fibrinogen concentration. This seems unlikely however, since the platelet count showed no significant differences between groups. Also, we do not routinely administer colloidal fluids in the postoperative period as some microsurgery units do, making hemodilution as reason for these changes even more unlikely. Also, an insufficient production or depletion of fibrinogen might lead to lower fibrinogen concentrations. Low levels of fibrinogen have been associated with bleeding in a variety of settings [20], [21], [22], [23], [24], [25], [26], [27], [28]. In addition, in many situations, the substitution of fibrinogen can often help to control the bleeding, leading to lower rates of blood transfusions [23], [26], [28], [29]. However, not only insufficient levels of fibrinogen and therefore an inadequate clot formation can cause bleeding, but also hyperfibrinolysis. Ongoing hyperfibrinolysis might also lead to a depletion of fibrinogen and therefore lower values. In such conditions, an increase in degradation products of fibrinogen is to be expected, which we did not measure in this study. Acute hyperfibrinolysis can also be assessed via rotational thromboelastometry and often be corrected by the administration of tranexamic acid, which binds to plasminogen, thus inhibiting its interaction with fibrinogen and preventing the dissolution of clots [30]. Both an insufficient clot formation as well as its untimely dissolution can therefore lead to clinically relevant bleeding, even despite meticulous surgical hemostasis. 

Based on these findings, patients with an inadequate post-operative increase of fibrinogen levels seem to be more likely to develop a relevant bleeding at the recipient but also at the donor site of free flap transfer. The resulting hematoma can compromise flap perfusion by an increased pressure on the microvascular pedicle or directly on the microvascular architecture of the flap. Also, such a bleeding often requires surgical re-exploration and the transfusion of blood products, which is associated with increased morbidity and cost and can further deteriorate the outcome of free tissue transfer [7], [8]. Therefore, the early identification of such patients as well as an early and targeted intervention seems desirable. Since the preoperative coagulation values did not differ significantly between groups, a close monitoring of changes in coagulation and especially fibrinogen levels seems warranted. In patients with an insufficient fibrinogen response and bleeding, the administration of fibrinogen concentrates should be discussed, especially if such a bleeding is refractory to surgical hemostasis. However, such interventions should be closely monitored to minimize the potential risk of (microvascular) thrombosis.

Several limitations apply to this study. First, the number of patients included is limited. Nonetheless, it represents one of the largest cohorts regarding this subject and, to our knowledge, the first study to depict changes in coagulation during the perioperative period in free tissue transfer. Also, despite the low number of patients developing postoperative bleeding, the differences between groups were statistically highly significant. Due to the retrospective nature and the limitation on a few key factors of coagulation, various influencing factors on the mentioned changes could potentially be missed. Even as fibrinogen and platelets only account for a part of the coagulation system, they represent the major influence on clot formation in the acute situation [31]. To take other influences into account as well as to assess not only the sole values of these parameters but also their function, advanced diagnostics like rotational thromboelastometry seems warranted in this context. 

## Conclusions

Free flap surgery induces acute and subacute changes in coagulation, comparable to other major surgeries and severe injuries. This leads to an increase in platelet count and fibrinogen over the post-operative course. These changes do not differ significantly between patients with an uneventful course and patients that develop thrombosis of the microvascular pedicle. However, patients that developed bleeding requiring surgical re-exploration showed an insufficient increase in fibrinogen, resulting in significantly lower fibrinogen levels. Therefore, monitoring and correction of fibrinogen levels might aid in preventing or treating bleeding complications following free flap surgery.
